# A Brazilian Population of the Asexual Fungus-Growing Ant *Mycocepurus smithii* (Formicidae, Myrmicinae, Attini) Cultivates Fungal Symbionts with Gongylidia-Like Structures

**DOI:** 10.1371/journal.pone.0103800

**Published:** 2014-08-07

**Authors:** Virginia E. Masiulionis, Christian Rabeling, Henrik H. De Fine Licht, Ted Schultz, Maurício Bacci, Cintia M. Santos. Bezerra, Fernando C. Pagnocca

**Affiliations:** 1 Instituto de Biociências, São Paulo State University, Rio Claro, SP, Brazil; 2 Museum of Comparative Zoology, Harvard University, Cambridge, Massachusetts, United States of America; 3 Department of Entomology, National Museum of Natural History, Smithsonian Institution, Washington, D.C., United States of America; 4 Section for Organismal Biology, Department of Plant and Environmental Sciences, University of Copenhagen, Copenhagen, Denmark; Emory University, United States of America

## Abstract

Attine ants cultivate fungi as their most important food source and in turn the fungus is nourished, protected against harmful microorganisms, and dispersed by the ants. This symbiosis evolved approximately 50–60 million years ago in the late Paleocene or early Eocene, and since its origin attine ants have acquired a variety of fungal mutualists in the Leucocoprineae and the distantly related Pterulaceae. The most specialized symbiotic interaction is referred to as “higher agriculture” and includes leafcutter ant agriculture in which the ants cultivate the single species *Leucoagaricus gongylophorus*. Higher agriculture fungal cultivars are characterized by specialized hyphal tip swellings, so-called gongylidia, which are considered a unique, derived morphological adaptation of higher attine fungi thought to be absent in lower attine fungi. Rare reports of gongylidia-like structures in fungus gardens of lower attines exist, but it was never tested whether these represent rare switches of lower attines to *L. gonglyphorus* cultivars or whether lower attine cultivars occasionally produce gongylidia. Here we describe the occurrence of gongylidia-like structures in fungus gardens of the asexual lower attine ant *Mycocepurus smithii*. To test whether *M. smithii* cultivates leafcutter ant fungi or whether lower attine cultivars produce gongylidia, we identified the *M. smithii* fungus utilizing molecular and morphological methods. Results shows that the gongylidia-like structures of *M. smithii* gardens are morphologically similar to gongylidia of higher attine fungus gardens and can only be distinguished by their slightly smaller size. A molecular phylogenetic analysis of the fungal ITS sequence indicates that the gongylidia-bearing *M. smithii* cultivar belongs to the so-called “Clade 1”of lower Attini cultivars. Given that *M. smithii* is capable of cultivating a morphologically and genetically diverse array of fungal symbionts, we discuss whether asexuality of the ant host maybe correlated with low partner fidelity and active symbiont choice between fungus and ant mutualists.

## Introduction

Mutualisms, symbiotic interactions between organisms in which each partner benefits, are widespread across the tree of life [1]. Many eukaryotes evolved obligate relationships with symbiotic organelles, such as mitochondria and chloroplasts, and provide stunning examples of ancient, evolutionarily stable mutualisms [2]–[4]. Co-evolutionary processes, reciprocal genetic changes in one species in response to changes in the partner species, shape these tight relationships, selecting for ecological specialization and resulting in co-diversification and eventually co-speciation [5]–[10]. Evolutionary patterns of co-speciation can be inferred secondarily from congruent phylogenetic histories (i.e., co-cladogenesis). Unfortunately, however, it is inherently difficult to study currently co-evolving organisms in order to understand the selective processes and proximate mechanisms underlying obligate interdependencies because currently observed patterns may not necessarily reflect the evolutionary interactions that shaped the symbiosis when it originated.

The complex symbiosis of fungus-growing ants with leucocoprineaceous fungi and other associated microorganisms provides a system that is well suited for studying the evolution of mutualistic interactions and the origins of fungiculture in insects [11]–[17]. The fungus-gardening ants of the tribe Attini comprise a monophyletic group of more than 250 described species [15], [18], [19] that are distributed throughout the New World from Argentina in the south to the United States in the north [20]–[23]. All fungus-growing ant species rely obligately on basidiomycete fungi that they cultivate for food [21], [24]–[26]. To enable the growth of the fungal symbionts, the ants provide nutrition to the fungus garden and prevent the growth of alien microorganisms [21], [27]–[31].

Originating around 50–60 million years ago, the ancestral attine agricultural system, i.e., “lower agriculture,” is practiced today by the majority of attine ant genera and species, which cultivate a closely related but poly- and paraphyletic group of leucocoprineaceous fungi [15], [32]. Around 20–30 million years ago, a particular lower-attine ant-fungus association gave rise to “higher agriculture,” which includes the well-known leafcutter ants [15]. The clade of fungi associated with higher attine ants is descended from a lower-attine fungal ancestor, and unlike the lower attine fungi, higher attine fungi are never found free-living apart from their ant hosts, suggesting strong co-evolutionary dynamics between higher attine ants and their cultivars [12], [27], [33]–[37]. The most significant morphological adaptations of higher attine fungi are the nutrient-rich hyphal tip swellings, the so-called gongylidia, which serve as the main food source for the ants and their brood [24], [26], [38]–[44].

In 1893, the pioneering mycologist Alfred Möller first described these hyphal tip swellings in the fungus gardens of *Acromyrmex coronatus*, which he called “Kohlrabikopf,” due to their morphological similarity to cabbage turnips (Möller [24], pp. 26). Later Wheeler [25] suggested the Hellenistic version of Möller’s term, “gongylidium, -a” (Greek = gongilis = turnip), for the same structure. In fungus gardens of higher attine ants, gongylidia occur in clusters, which Möller [24] termed “Kohlrabihäufchen,” and Weber [38] later called “staphyla, -ae” (Greek = cluster of grapes). Chemical analyses showed that gongylidia contain glucose, mannitol, trehalose, glycan, arabinitol, and glycogen, in addition to lipids, and ergosterol [26], [40], [45], [46], as well as free amino acids [34], [45]. In contrast, the filamentous hyphae contain high protein concentrations but only low concentrations of lipids and carbohydrates [26], [45]. In addition, a recent study demonstrated how the co-evolutionary adaptations of specific laccase enzymes, which are highly expressed in the gongylidia, participate in the detoxification of secondary plant compounds present in the leaf material [47].

The presence of gongylidia in the fungus gardens of higher attine ants is considered an exclusively co-evolved, mutualistic adaptation and gongylidia are not known to convey any fitness benefits to the fungus outside the ant symbiosis [13], [15], [21], [35], [37], [48]. Here we report the occurrence of gongylidia in fungus gardens of the asexual lower attine ant *Mycocepurus smithii* from Brazil. *M. smithii* is distributed across tropical and subtropical habitats in Central and South America [49], [50] and consists of a mosaic of sexually and asexually reproducing populations [50]. Populations from southeast Brazil were found to reproduce strictly asexually via thelytokous parthenogenesis [50], [51]. Interestingly, *M. smithii* was previously reported to be one of the very few attine ant species to cultivate a genetically diverse array of fungi [13], [32], [52]. In contrast, some other lower attine species are known to be faithful to a single cultivar lineage [17], [53]. In this study, we test whether *M. smithii* cultivates leafcutter ant cultivars or, alternatively, whether lower attine cultivars are capable of producing gongylidia. In addition, we explore the hypothesis of whether asexual reproduction in *M. smithii* may have enabled and/or prompted the cultivation of morphologically and genetically diverse cultivars.

## Materials and Methods

### Study site and field observations

**Figure 1:**
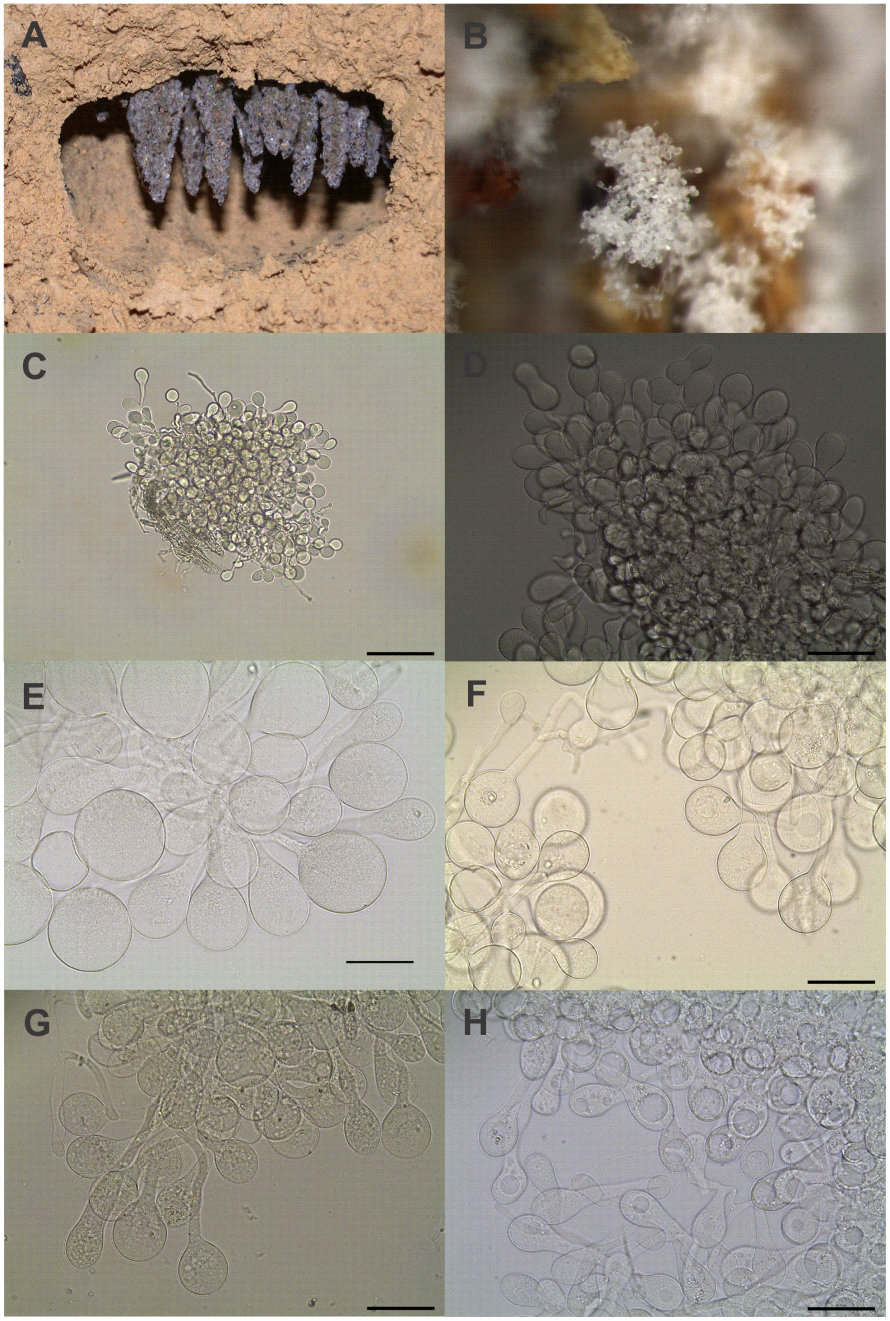
Cultivated fungi and gongylidia observed in gardens of *Mycocepurus smithii* (A, B, C, D) and some species of higher Attini (E, F, G, H). (A) Nest chamber and pendant fungus garden of *Mycocepurus smithii*; (B) gongylidia organized in staphylae in the fungus garden of *M. smithii* (8x magnification); (C) staphylae in a *M. smithii* cultivar. Gongylidia in the fungus gardens of *M. smithii* (D), *T. fuscus* (E), *Ac. disciger* (F), *A. laevigata* (G), and *A. sexdens* (H). The scale bar in figure C represents 100 µm; scale bars in figures D, E, F, G, and H represent 50 µm.

During a field class taught at São Paulo State University in Rio Claro, Brazil (22.3955°S, 047.5424°W; elevation 608 m), we excavated nests of multiple fungus-growing ant species to illustrate their natural histories and the intricate symbiosis between ants, fungi, and other associated microorganisms. Nest excavations followed the methodology described earlier [51], [54]. We excavated a total of three *M. smithii* fungus chambers, which received the following collection codes: CR110715-01, CR110715-02, and CR110718-01. Fungus-chamber CR110715-01 was found at 25 cm depth. It was 2.5 cm wide and 2 cm high and contained a pendant fungus garden hanging from the chamber ceiling (Fig. 1A), multiple workers, no brood, and no queen. The second chamber, CR110715-02, was located directly underneath the first chamber at 53 cm depth, was slightly larger with a diameter of 3 cm and contained a pendant fungus garden, a single queen, multiple workers, and no brood. We assume that these two chambers belonged to the same nest. The third *M. smithii* fungus chamber, CR110718-01, was excavated approximately 50 m distant from the first nest. Only a single chamber was encountered at 50 cm depth. It was 5 cm wide and 4 cm high, contained a pendant fungus garden, eleven workers, and neither a queen nor brood. The sizes of the fungus chambers and their locations in the soil are consistent with results reported from other *M. smithii* populations in Latin America [51], [54], [55]. The live ant colonies and their fungus gardens were collected with a surface-sterilized spoon and placed into a laboratory nest for further observation. The lab nest consisted of a plastic container with a plaster of Paris bottom; see reference [56] for lab nest setup. Ants were identified using a Leica MS5 stereomicroscope and voucher specimens were deposited in Maurício Bacci’s Molecular Evolution Laboratory at São Paulo State University in Rio Claro. Collections were conducted under collecting permit number 14789-3 issued by the Ministério do Meio Ambiente – MMA and the “Instituto Chico Mendes de Conservação da Biodiversidade “ (ICMBio).

### Macro and microscopic observations

The presence of staphylae in *M. smithii* gardens was first noted during observations of the live colonies with a Leica MZ16 stereomicroscope and confirmed under higher magnification with a Leica DM750 bright-field microscope. The fungus garden of colony CR110715-02 was maintained in a dark room at 25°C for two days and measurements of gongylidia, which were grouped into staphylae, were taken with the Leica Application Suite V3 software package under a Leica DM750 bright-field microscope. For microscopic studies, we removed the staphylae from the fungus garden with a pair of acupuncture needles, placed them on a microscope slide, and submerged them in a drop of 15% glycerin solution. Staphylae were collected and 40 gongylidia were measured from a fungus garden of each of the following: lab colonies of *Atta sexdens*, *A. laevigata*, and *Acromyrmex disciger,* and a field-collected colony of *Trachymyrmex fuscus*. The colonies were collected on the campus of São Paulo State University in Rio Claro between October 2009 and September 2011.

The normality of the morphological measures was tested using Shapiro-Wilk’s test and the homogeneity of variance was assessed with Levene’s test of homoscedasticity. To test for variance of size distribution in gongylidia of different fungi, we conducted an analysis of variance (one-way ANOVA and Tukey test) with a significance level of less than 1% (p<0.01) using the software package BioEstat 5.0 [57].

### Genotyping and molecular phylogenetic analyses

To genotype the gongylidia-bearing *M. smithii* cultivar CR110715-02, we extracted genomic DNA from tissue samples of the staphylae following the methodology described in Martins Jr. et al. [58]. The ITS region was amplified according to Manter and Vivanco [59] using the forward primer ITS 5 (5′-GGAAGTAAAAGTCGTAACAAGG-3′) and reverse primer ITS 4 (5′-TCCTCCGCTTATTGATATGC-3′) [60]. The PCR reaction consisted of an initial 2-min incubation at 96°C, followed by 28 cycles of 46 s at 96°C, 30 s at 50°C and 4-min at 60°C. The PCR product was gel-purified using an Illustra™ GFX™ PCR DNA and Gel Band Purification Kit (GE Healthcare, UK). The same primer pair was utilized for amplification and sequencing. The sequencing reaction was prepared with 100 ng of PCR template, 6 pmols primers, 2.0 µl BigDye Terminator (Applied Biosystems), 1.0 µl buffer (200 mM Tris.HCl, 5 mM MgCl_2_) and ddH_2_O. Sequencing products were purified and then analyzed on an automated sequencer ABI3500 (Applied Biosystems). The consensus sequence was edited with the program BIOEDIT 7.0.5 [61] and aligned using CLUSTALW [62]. The obtained sequence was deposited at NCBI’s GenBank (www.ncbi.nih.gov) as accession number JX027477 and was compared to other ITS sequences deposited in GenBank. The resulting ITS DNA sequence consisted of 606 base pairs and nucleotides at positions 76 and 514 bp were unknown. The ITS DNA sequence of *M. smithii* cultivar CR110715-02 is identical to ITS sequences of *M. smithii* cultivars from Panama, Costa Rica, and Trinidad; *M. tardus* from Panama; *Myrmicocrypta ednaella* from Panama; and a new *Mycocepurus* species from Guyana (erroneously identified as *M.* cf. *goeldii*), all of which were previously published in Mueller et al. [13] and Kellner et al. [63].

To determine the molecular phylogenetic placement of the gongylidia-bearing *M. smithii* fungus, we added this sample to a data matrix of attine and free-living leucocoprineaceous fungal ITS DNA sequences consisting of 305 fungal taxa and 1042 nucleotide sites (including indels), which was previously published by Mehdiabadi et al. [17]. Sequences were aligned in MAFFT v7.017 [64] using the E-INS-I algorithm, a 200PAM/k = 2 scoring matrix, a gap opening penalty of 1.53, and an offset value of 0.

#### Maximum likelihood analyses

Initially, a best-fit model of sequence evolution was selected for the entire unpartitioned fungal ITS alignment under the Akaike information criterion as calculated in jModel Test 2.1.1 [65]. Using the resulting model, GTR+I+G (with 6 rate categories), an initial ML “best tree” analysis was conducted in Garli 2.0.1019 [66] consisting of 100 replicates using parallel processing and default parameter values. The results of this initial analysis were used to divide the sequence data into faster- and slower-evolving character sets. This was done by evaluating character evolution on the ML tree in MacClade 4.08 [67] under the parsimony criterion and assigning characters requiring 3 or fewer steps to a “slow” partition (596 characters) and characters requiring 4 or more steps to a “fast” partition (446 characters). Each of these two partitions was fit, again using the AIC in jModel Test, to an evolutionary model. Based on the results, a second “best tree” search was conducted with two partitions, a “slow” partition under the JC model and a “fast” partition under the HKY+G model, consisting of 100 searches and deviating from the default settings as follows: topoweight = 0.01; brlenweight = 0.002. ML bootstrap analyses in Garli, also employing the same two partitions and models, consisted of 1000 pseudoreplicates, deviating from default settings as follows: genthreshfortopoterm = 5000; scorethreshforterm = 0.10; startoptprec = 0.5; minoptprec = 0.01; numberofprecreductions = 1; treerejectionthreshold = 20.0; topoweight = 0.01; brlenweight = 0.002.

#### Bayesian analyses

The fungal alignment was analyzed under Bayesian criteria as implemented in MrBayes v3.2.1 [68] with the two partitions and models described above and with 10 million generations and samplefreq = 1000. All parameters were unlinked across partitions except for branch-lengths and topology. All analyses were carried out using parallel processing (one chain per CPU) with nucmodel = 4by4, nruns = 2, nchains = 8, and samplefreq = 1000. To address known problems with branch-length estimation in MrBayes, we reduced the branch-length priors using brlenspr = unconstrained:Exp(100) based on the procedure suggested in Brown et al. [69] and as applied in Ward et al. [70] and Rabeling et al. [50]. Burn-in and convergence were assessed using Tracer v1.5 [71], by examining potential scale reduction factor (PSRF) values in the MrBayes.stat output files, and by using Bayes factor comparisons of marginal likelihoods of pairs of runs in Tracer, which employs the weighted likelihood bootstrap estimator of Newton and Raftery [72] as modified by Suchard et al. [73], with standard error estimated using 1,000 bootstrap pseudoreplicates.

## Results

**Figure 2:**
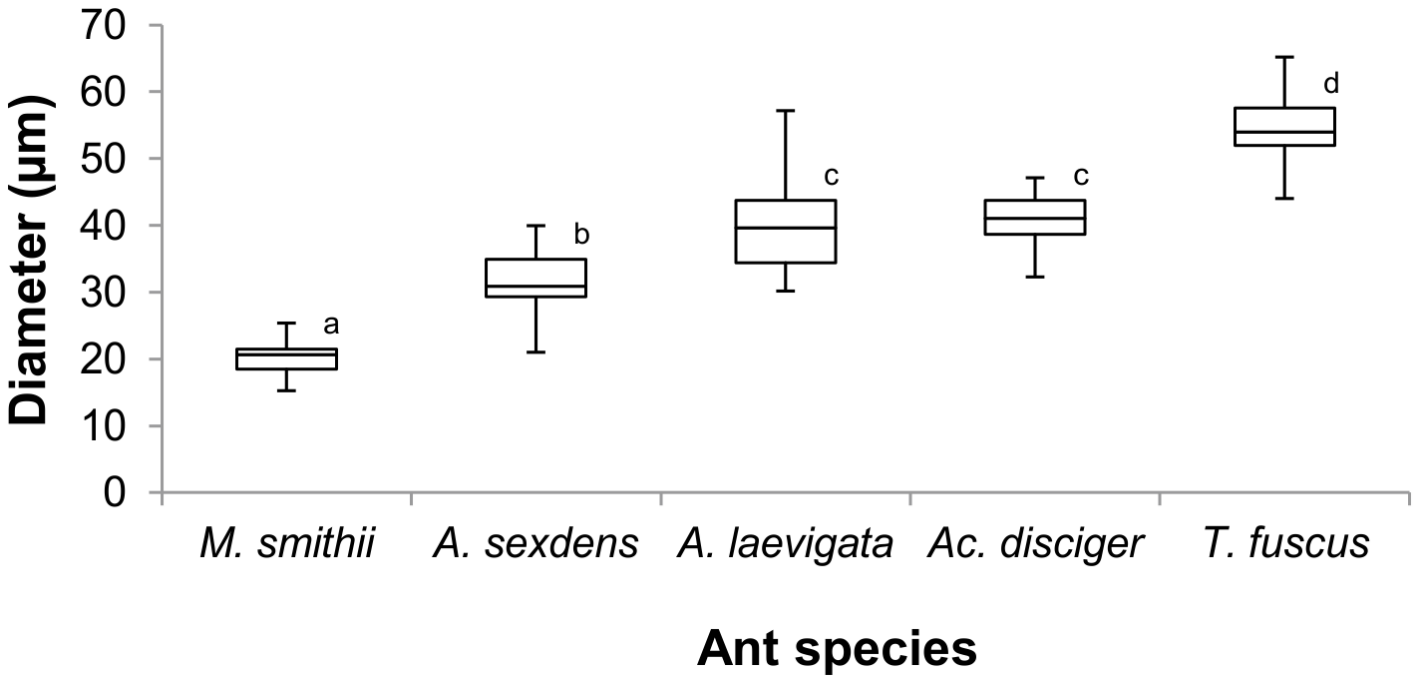
Comparison of gongylidia diameter from fungi cultivated by *M. smithii* and four species of higher Attini. The diameter of each gongylidium (n = 40 gongylidia per colony per ant species) was measured at its widest point. Mean values that are annotated with different letters are significantly different from each other (one-way ANOVA and Tukey test, p<0.01).

The fungal cultivars collected from three fungus chambers belonging to at least two *M. smithii* colonies contained gongylidia-like structures that were organized in staphylae (Figs. 1B–D). Our microscopic examination at 400x magnification showed that the gongylidia of this *M. smithii* fungal cultivar were morphologically very similar to the gongylidia found in fungal cultivars of *T. fuscus*, *Ac. disciger*, *A. laevigata*, and *A. sexdens* (Fig. 1 E,F,G,H), differing only in their smaller size (Fig. 2).

The diameter of gongylidia found in the *M. smithii* garden varied between 16.3 µm and 25.41 µm, which was significantly smaller than the gongylidia diameters in the studied *Trachymyrmex*, *Acromyrmex,* and *Atta* species (one-way ANOVA and Tukey test, p<0.01; Fig. 2). Gongylidia diameter in the higher attine cultivars was distributed along a continuum, on which *A. sexdens* gongylidia were the smallest, ranging from 21.04 µm to 39.99 µm in diameter, and on which *T. fuscus* gongylidia were the largest, ranging between 42.01 µm and 68.26 µm in diameter. The cultivars of all five examined ant species had gongylidia diameters significantly different from one another, except for *Ac. disciger* and *A. laevigata* (Fig. 2).

Gongylidia are thought to be highly specialized morphological adaptations of higher attine cultivars. The detection of gongylidia in the fungus garden of one of the most “primitive” attine lineages therefore suggests at least three alternative explanations: (i) *M. smithii* is capable of growing leafcutter ant cultivars; (ii) gongylidia production arose independently in the cultivars of lower and higher attine ants; or (iii) gongylidia are ancestrally present in at least some lower attine fungi, including, presumably, the lineage that gave rise to higher attine fungi. To distinguish between these evolutionary scenarios, we conducted molecular phylogenetic analyses of the ITS region of attine cultivars to determine the phylogenetic position of the gongylidia-producing *M. smithii* fungus.

**Figure 3:**
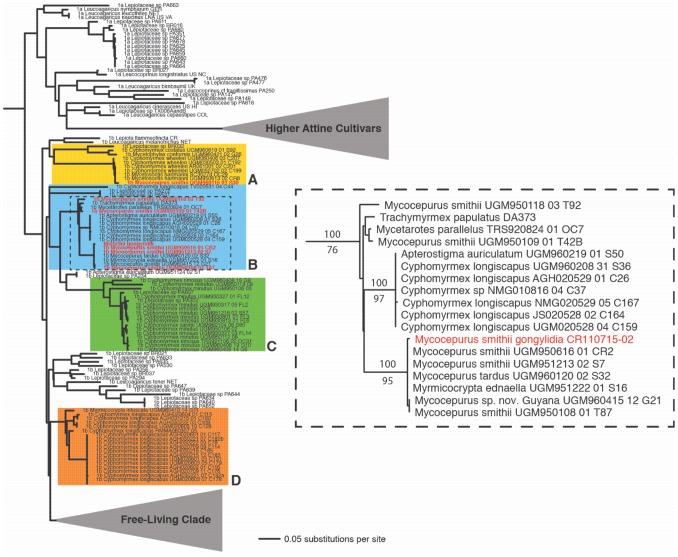
ITS phylogeny of Clade 1 of the fungal tribe Leucocoprineae. Clade 1 of the Leucocoprineae includes four primary clades of attine cultivars and closely related free-living fungi, here indicated as subclades A–D (sensu Mehdiabadi et al. [17]). The clade within subclade B that contains the gongylidia-bearing fungal cultivar of *M. smithii* from Rio Claro is indicated by dashed lines and is enlarged in the inset. Within the inset, the name of the gongylidia-bearing cultivar is indicated in red. The names of other cultivars of *M. smithii* in subclades A and B are indicated in red; *M. smithii* additionally cultivates fungi in Clade 2 (not shown here; see Mehdiabadi et al. [17]). The phylogram results from maximum-likelihood analyses of ITS sequence data of 305 fungal taxa. In the inset, numbers above branches are Bayesian posterior probabilities; numbers below branches are maximum-likelihood bootstrap proportions.

Both Bayesian and maximum likelihood phylogenetic analyses agree that the gongylidia-bearing *M. smithii* fungal cultivar of the Rio Claro population is embedded in the so-called “Clade 1” of the fungus tribe Leucocoprineae (Fig. 3) and is sequence identical with fungi cultivated by *M. smithii* (from Trinidad, Panama, Costa Rica), *M. tardus* (Panama), an undescribed species of *Mycocepurus* from Guyana, and *Myrmicocrypta ednaella* (Panama) (MLBP = 95, BPP = 100). Interestingly, a clade only a couple of nodes removed from the gongylidia-producing *M. smithii* cultivar contains a fungus cultivated by *T. papulatus* (Fig. 3), which is a member of the higher Attini. The highly derived higher attine fungal clade, which includes the cultivars of the leafcutter ants, is shown in our analysis to have arisen from a lower-attine fungal ancestor in Clade 1, which is consistent with earlier results [12], [16].

## Discussion

Although somewhat smaller in diameter, the gongylidia found in the fungus gardens of the asexual fungus-growing ant *M. smithii* occurring in Rio Claro are similar in shape to gongylidia found in the cultivars of higher attine species. This is surprising because gongylidia are thought to be a derived morphological specialization in higher attine fungi that originated as a result of the ant-fungus co-evolution that produced the higher attine ants, including the leafcutter ant genera *Atta* and *Acromyrmex* [14], [21], [48]. The fact that gongylidia were encountered in three fungus gardens of at least two separate nests suggests that gongylidia may occur frequently in *M. smithii* fungus gardens in this population in Rio Claro. Unfortunately, the frequency of occurrence of gongylidia in the *M. smithii* study population is currently unknown and will only be determined by a comprehensive survey across populations and seasons. It should be noted here that we have never observed gongylidia in *M. smithii* gardens previously, although we have excavated and inspected cultivars from dozens of *M. smithii* nests in Central and South America (Bacci, Rabeling, Schultz, unpublished data). One of us (TRS), however, recalls observing in 1993 what appeared to be staphylae in the garden of a lab colony of a lower attine ant, probably *Mycetosoritis hartmanni*.

In general, accounts of gongylidia-like structures in the fungus gardens of lower attines are rare in the Attini literature. Möller [24] was the first to provide a detailed account of the fungi cultivated by leafcutter ants and by lower attine species and reported hyphal swellings from gardens of *Apterostigma wasmannii* and to a lesser extent from fungus gardens of *Cyphomyrmex strigatus*. Möller [24] clearly distinguished these hyphal swelling from the gongylidia and staphylae of the leafcutter ants, however. Paraphrasing Möller’s original account ([24], pp.108), he reported that the globular and densely packed gongylidia of the leafcutter ants represented the most “perfect” food bodies, whereas the gongylidia of *A. wasmannii* were tubular in shape and less densely aggregated in the staphylae. Möller [24] further distinguished the fungal cultivars of *Acromyrmex* and *Apterostigma* experimentally, demonstrating that only leafcutter fungi continued to produce gongylidia in culture medium, whereas the *A. wasmannii* and *C. strigatus* cultivars reverted invariably to the characteristic filamentous mycelial growth. Unfortunately, we were not able to culture the *M. smithii* fungus, but its morphology was significantly different from the tubular hyphal swellings of *Apterostigma* and *Cyphomyrmex* fungi observed by Möller (compare Figs. 26 & 27 in [24]).

It remains uncertain whether the tubular swellings in *Apterostigma* and *Cyphomyrmex* gardens observed by Möller, the gongylidia of higher attine cultivars, and the gongylidia of the *M. smithii* cultivar reported here are developmentally homologous. Interestingly, the developmental origins of gongylidia remain entirely uninvestigated. One hypothesis suggests that gongylidia are modified cystidia, the hyphal swellings found in the hymenium of many free-living basidiocarps (J.A. Scott pers. obs., cited in [39]) and a hypothesis of the origin of ant fungivory suggests that “proto-gongylidia” may have served as the fungal analogue of ant-attractant elaiosomes of plant seeds, which provide a food reward in return for ant dispersal [14], [24].

The mean diameter of the gongylidia in the *M. smithii* cultivar was smaller than the mean diameter of gongylidia of higher attine species collected in Rio Claro. Earlier mycological studies by Möller [24] on the fungus gardens of *Acromyrmex coronatus* and *Ac. disciger* reported relatively small gongylidia sizes (10–24 µm), which are comparable in size to those found in *M. smithii* gardens. *Ac. disciger* was also studied by us and the fungi from the Rio Claro population had significantly larger gongylidia than those from Blumenau reported by Möller [24]. Gongylidia size could differ significantly between colonies of the same ant species because the same species may utilize different cultivar species or strains in different nests and in different geographic locations. Gongylidia size could also differ in the same cultivar strain depending on the developmental status of the ant colony and the amount of nutrition provided by the ants.

The mechanisms underlying the production of gongylidia and the factors determining their size remain poorly understood. Previous studies of staphylae in higher attine nests suggested that the ants’ constant pruning stimulated the formation of gongylidia [74], [75]. In addition, Powell and Stradling [76] showed that the quality of the substrate, the pH, and the temperature affect the growth and size of gongylidia in cultivars of three higher attine species (*A. cephalotes*, *Ac. octospinosus,* and *T. urichi* from Trinidad) when cultivated *in-vitro* on agar plates. A recent genetic expression study added a functional dimension to our understanding of gongylidia by showing that one laccase enzyme, which is highly expressed in the gongylidia of leafcutter ants, digests plant defensive phenolic compounds [46]. The ability to up-regulate laccase expression in the presence of fresh leaf material is thought to be a pre-adaptation of the fungus that evolved prior to the symbiosis with attine ants [46].

The phylogenetic position of the gongylidia-producing *M. smithii* cultivar in Clade 1 of the Leucocoprinae phylogeny, the uncertainty surrounding the phylogenetic position of the higher attine fungi within Clade 1, and the lack of information regarding the distribution of gongylidia in lower attine fungi leave open the question of whether gongylidia evolved repeatedly in leucocoprineaceous fungi or whether they had a single origin. The fungal phylogeny further shows that *M. smithii* cultivates a variety of genetically distant cultivars from both leucocoprineaceous Clades 1 and 2, which is consistent with earlier studies [13], [17], [32], [52]. Fungus-growing ants and their fungal cultivars are hypothesized to have evolved largely via diffuse co-evolution, in which groups of species of ants and fungi interact [16]. However, particular ant and fungal species in the *Cyphomyrmex wheeleri* group were shown to have been exclusively associated over long periods of time, potentially for millions of years [17]. The opposite pattern is observed in *M. smithii*, which is capable of cultivating a high diversity of fungal species in both Clades 1 and 2. It has been hypothesized that this pattern of fungal association indicates that symbiont choice behavior (e.g., choosing cultivars adapted for particular microhabitats) is more important for *M. smithii* colonies than being faithful to a specific cultivar type (i.e., high partner fidelity) [63]. In the absence of a cultivar/habitat correlation, the observation that *M. smithii* cultivates an unprecedented genetic variety of cultivars is also consistent with weakened symbiont choice. *M. smithii* could only have acquired such a high diversity of cultivars by frequently domesticating free-living cultivars *de novo* and/or by acquiring novel cultivars from sympatric fungus-growing ants. A local study of Panamanian *M. smithii* colonies and their associated fungi hypothesizes that, because ants and fungi reproduce asexually and accumulate deleterious mutations (according to Muller’s ratchet), the ant’s frequent acquisition of novel cultivars functions as a kind of recombination, purging the ants from “deleterious fungi” and vice versa [63], [77]. It remains to be demonstrated that the fungi cultivated by *M. smithii* suffer from Muller’s ratchet because the large feral populations from which the cultivars are domesticated experience frequent sexual recombination [13], [32].

An alternative null hypothesis is that, with regard to lower attine fungi, patterns of ant-fungus association in *M. smithii* are random. One hypothesis that would predict such randomness is that, due to the lack of genetic recombination, the ants’ genomes accumulate deleterious mutations, some of which affect their olfactory receptors. Inclusive fitness theory predicts that conflict over reproduction is absent in a clonal society. Thus, nestmate recognition and mate recognition are expected to become obsolete in an asexually reproducing species such as *M. smithii* and, if olfaction genes are not actively maintained by natural selection, these genes will accumulate non-synonymous mutations. Consequently, the ants’ ability to recognize nestmates, or a specific fungal symbiont for that matter, may then deteriorate, and the ants may become unable to distinguish between cultivar types. In contrast, sexually reproducing attine species in the genus *Cyphomyrmex* have been experimentally shown to prefer the fungal cultivars they grew up with [78]. If olfactory abilities deteriorate in asexual fungus growing ants, which still needs to be demonstrated, the predicted outcome would be congruent with the pattern we observe in nature, in which an asexual fungus-growing ant cultivates a wide variety of morphologically and genetically different fungi. Future studies will first test whether the genes involved in olfaction are under relaxed natural selection in asexual populations of *M. smithii* and, second, comprehensively analyze the co-evolutionary interactions between *M. smithii* and its fungal cultivars on a global scale (Rabeling, Bacci, Schultz, unpublished data).
